# Discrimination of cell cycle phases in PCNA-immunolabeled cells

**DOI:** 10.1186/s12859-015-0618-9

**Published:** 2015-05-29

**Authors:** Felix Schönenberger, Anja Deutzmann, Elisa Ferrando-May, Dorit Merhof

**Affiliations:** 1 Institute of Imaging & Computer Vision, RWTH Aachen University, Templergraben 55, Aachen, 52074 Germany; 2 Bioimaging Center (BIC), University of Konstanz, Universitätsstraße 10, Konstanz, Germany; 3 Stanford University School of Medicine, Division of Oncology, 269 Campus Drive, Stanford, 94305 CA USA

**Keywords:** Classification, Image analysis, Feature selection, Cell cycle phases

## Abstract

**Background:**

Protein function in eukaryotic cells is often controlled in a cell cycle-dependent manner. Therefore, the correct assignment of cellular phenotypes to cell cycle phases is a crucial task in cell biology research. Nuclear proteins whose localization varies during the cell cycle are valuable and frequently used markers of cell cycle progression. Proliferating cell nuclear antigen (PCNA) is a protein which is involved in DNA replication and has cell cycle dependent properties. In this work, we present a tool to identify cell cycle phases and in particular, sub-stages of the DNA replication phase (S-phase) based on the characteristic patterns of PCNA distribution. Single time point images of PCNA-immunolabeled cells are acquired using confocal and widefield fluorescence microscopy. In order to discriminate different cell cycle phases, an optimized processing pipeline is proposed. For this purpose, we provide an in-depth analysis and selection of appropriate features for classification, an in-depth evaluation of different classification algorithms, as well as a comparative analysis of classification performance achieved with confocal versus widefield microscopy images.

**Results:**

We show that the proposed processing chain is capable of automatically classifying cell cycle phases in PCNA-immunolabeled cells from single time point images, independently of the technique of image acquisition. Comparison of confocal and widefield images showed that for the proposed approach, the overall classification accuracy is slightly higher for confocal microscopy images.

**Conclusion:**

Overall, automated identification of cell cycle phases and in particular, sub-stages of the DNA replication phase (S-phase) based on the characteristic patterns of PCNA distribution, is feasible for both confocal and widefield images.

## Background

Many physiological processes in an eukaryotic cells are influenced by the cell cycle. Assigning cellular events to specific cell cycle phases allows to detect subtle phenotypes that only become manifest periodically at certain stages of the cell cycle. Establishing a link between these phenotypes and the cell cycle phase leads to a better understanding of the underlying cellular processes.

There are different ways to monitor how cells pass through different phases of the cell cycle. A widely used approach is to label single proteins that show a cell cycle specific behavior. The amount or distribution of such a protein usually depends on the cell cycle phase. This information can be used to classify cells and assign them to different phases. A protein with cell cycle dependent properties is the proliferating cell nuclear antigen or PCNA. This protein is a processivity factor for DNA polymerase in eukaryotic cells. It enhances the binding of the polymerase to the DNA template and enhances DNA synthesis. During the S (synthesis)-phase of the cell cycle it localizes to sites of active replication.

**Fig. 1:**
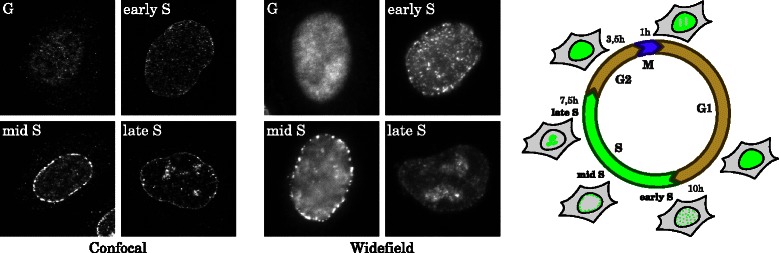
Distribution patterns of PCNA in the cell nucleus in confocal (left) and widefield (middle) microscopy images. Schematic outline of the cell cycle with characteristic, phase-dependent distribution of PCNA in the nucleus (right)

It has been shown that PCNA is suitable to discriminate three different stages of S phase cells (early, mid and late S phase) [1–3]. Representative images of cells in these three different stages as well as in the G phase, which has a similar pattern of PCNA localization, are shown in Fig. 1 (left, middle). The microscopy images were acquired both at a confocal and a widefield microscope.

The motivation of this work is to provide a general and extensible framework enabling the automatic recognition of different phases of the cell cycle, with special interest in detecting early, intermediate and late stages of S phase in fixed cells based on the distribution of PCNA in fluorescence images. Fixed cells allow the use of antibody staining which simplifies sample handling and image acquisition and is therefore widely applicable. However, as compared to the analysis of live cell imaging [4, 5] which provides additional temporal information, the classification of fixed specimen is more challenging from an computational point of view. For this reason, sophisticated algorithms for reliable automated cell cycle classification are required, which are able to address variable fluorescence patterns, changes in SNR and shape as well as touching cells.

The contributions of this work comprise a processing pipeline for the discrimination of cell cycle phases in PCNA-immunolabeled cells, an in-depth analysis and selection of appropriate features for classification, an in-depth evaluation of different classification algorithms, as well as a comparative analysis of classification performance achieved with confocal versus widefield microscopy images.

## Biological background - cell cycle

Eukaryotic cells multiply through a process called cell division. Before a cell can divide it has to grow in size, duplicate its whole genetic material exactly once and distribute the chromosomes between the daughter cells. After cell division has completed each daughter cell must contain the original genetic information. The underlying molecular events are precisely orchestrated in time and space. The entire process between two cell divisions is termed cell cycle. The cell cycle can be subdivided in different cell cycle phases (Fig. 1, right). In the first phase of the cell cycle (G1 phase) the cell grows and prepares for DNA replication by synthesizing proteins which are needed in subsequent cell cycle phases. In the following phase (S phase) the DNA is duplicated. After successful DNA duplication, the cell enters the next phase (G2 phase) and prepares for cell division. During mitosis (M phase) two identical sets of chromosomes are distributed at exactly opposed sides of the equatorial plane. At the end of mitosis, two daughter cells form both starting the cell cycle again.

In both G phases (G1, G2), PCNA is equally distributed over the whole nucleus. During DNA replication in S-phase it marks sites of DNA synthesis, which spread through the genome in a characteristic temporal pattern. In the early S phase, PCNA agglomerates to small, equally distributed foci. In the mid S phase these foci are located at the nuclear periphery, and in the late S phase there are large foci near the center of the nuclei. In mitosis (M), PCNA is displaced from the condensed chromosomes, resulting in areas devoid of protein.

## Methods

In this section, the image acquisition and image processing methods for discriminating cell cycle phases in cells labeled with PCNA-specific antibodies are presented. Section ‘Image acquisition’ describes the procedure for immunofluorescence staining and biological image acquisition. Section ‘Preprocessing’ details the preprocessing steps taken in order to segment individual cells for further processing. In Section ‘Features’ the features used to classify the cell cycle phases are presented. Finally, the classification process itself, with a set of appropriate classifiers is presented in Section ‘Classification’.

### Image acquisition

For immunostaining of PCNA, 1×10^5^ HeLa Kyoto cells were grown in 12-well dishes containing glass coverslips. After 24 h of growth, cells were fixed in pre-chilled (−20 °C) methanol and acetone for 5 min and 1 min, respectively. After washing in PBS, unspecific binding was blocked by incubating in PBS containing 1 % bovine serum albumin (Sigma) for 30 min at room temperature. Immunolabeling with primary anti-PCNA antibody (rabbit polyclonal, abcam, ab18197, 1:500) was performed overnight at 4 °C in a humidified dark chamber. After washing in PBS, bound primary antibody was labeled with fluorophore conjugated secondary antibody for 1 h at room temperature using Alexa Fluor 488 antibody (goat polyclonal, Molecular Probes; A-1108, 1:400). Antibody dilutions were made in PBS containing 10 % normal goat serum (Sigma). Unbound secondary antibody was removed by washing in PBS, cell nuclei were counterstained with Hoechst 33342 (Invitrogen) and mounted on a microscope slide using Aqua-Poly/Mount (Polysciences).

All fluorescence images were acquired using either a widefield fluorescence microscope (Cellobserver HS, Zeiss) or a confocal laser scanning microscope (LSM 510 Meta, Zeiss), both equipped with a 40 x objective lens (LD Plan-Neofluoar, Zeiss).

### Preprocessing

The preprocessing steps comprise a segmentation method (Section ‘Segmentation’) to identify the regions of interest in an image, followed by a cluster splitting method (Section ‘Cluster splitting’) used to refine such a segmentation to get better results in cases of cell clusters.

#### Segmentation

In a first step, it is necessary to segment the image, i.e. distinguish the cell nuclei from the background. For a general review about cell segmentation, please refer to [6].

Both the widefield and confocal microscopy images considered in this work show cell nuclei that are well separated from the background. For this reason, entropy-based thresholding is applied for segmentation, which is particularly suited to process images which have a well-defined background, but may vary in overall brightness. Entropy-based thresholding methods operate in two alternative ways: They either try to maximize the entropy of the thresholded image meaning that the thresholded image contains a maximum of information, or minimize the cross entropy between input and thresholded image, which preserves the information. In this work, we employ Li entropy thresholding [7].

### Cluster splitting

One of the problems encountered regularly when segmenting biological images are cell clusters, i.e. individual cells are not well separated and clusters of several cells are identified as single objects. If the image contains touching or overlapping cells it is nearly impossible to get a perfect segmentation straight away. Instead, a cluster splitting method is applied after the initial segmentation procedure to refine the segmentation. In the literature, various approaches for cell cluster splitting have been proposed, which are reviewed in [8].

In general, there are two classes of methods for cell cluster splitting, marker-controlled watershed and geometric methods:

Cluster splitting via the watershed method [9, 10] is commonly applied to split concave objects in binary images. It is based on the watershed segmentation algorithm, which is applied to the Euclidean distance map of the binary segmentation. The watershed algorithm works very well on circular objects, however, it is very sensitive to segmentation artifacts. Fringy segmentation borders or slight undersegmentation will cause the watershed method to split single nuclei, resulting in an oversegmentation.

**Fig. 2:**
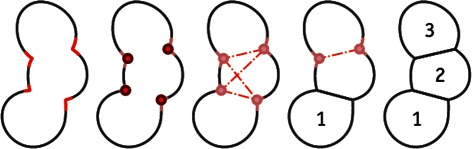
Schematic outline of geometric cluster splitting

Geometric methods take advantage of geometric properties, such as convexity or radial-symmetry of a cell, and are less prone to oversegmentation. For this reason, a geometric cluster splitting method [11] is employed in this work. As shown in Fig. 2, this geometric approach splits an object between two concave points on the segment outline. First, possible split points are identified as curvature maxima on the contour. From this set of split points, possible split hypotheses are derived and evaluated with a cost function. The construction of split hypotheses is based on the following constraints introduced in [11]: Split points are grouped by an anti-parallel constraint to ensure that pairs of split points are not on the same side of the object. The non-intersection constraint holds if the hypothesized segments do not intersect, and the convexity constraint requires each hypothesized segment to be convex. Since the nuclei of HeLa cells used in this work have a very similar size and shape, we extended this geometric cluster splitting and also consider the size of the cluster. Our additional size constraint ensures that splits are only performed on clusters with a minimal size. The cluster splitting itself is an iterative process: For increasing *n*, starting with *n*=2, split hypotheses with *n* segments are constructed and the segmentation with the lowest cost function is chosen. Finally, the size of the resulting split objects is considered and only cell nuclei within the desired size range are kept. The lower boundary of this range allows sorting out small objects and dead, shrunken cells. The upper boundary allows to detect invalid clusters that cannot be split, which may occur if the cells are packed very densely so that no significant curvature maxima are present to separate them.

### Features

This section introduces the features (computed on individual, segmented cells) used to discriminate the cell cycle phases. A feature is a real value calculated from the intensity levels or the extracted contour of a specified region of interest (ROI), describing a certain property of this region. All features are inserted into a feature vector. In order to obtain an appropriate description of the respective real world object, the feature vector has to collect a wide range of properties. The employed features must allow the differentiation of objects from different classes, but should show only little differences between representatives of the same class. For the purpose of differentiating cell cycle phases, features that are invariant to location, scale and rotation are required.

This work uses a variety of features to capture the properties of the PCNA spots inside the nuclei. In the following, two big classes of features, namely histogram features and Haralick texture features, are presented.

#### Histogram features

Histogram features are features which are derived from the histogram of an image. On floating point images or images with a higher bit depth, the intensity levels are binned. As a consequence, the histogram is less accurate but becomes manageable. From a histogram, several statistical values, suchs as mean, standard deviation, skewness and kurtosis, can be derived. The mean value can be used e.g. to distinguish between bright foci and the darker rest of the nucleus. In combination with the polar image (Section ‘Polar images’) of a segmented cell, which is further divided into columns (in the following referred to as zones), a feature vector containing the mean values of all zones can be seen as location distribution of the PCNA foci.

#### Histogram of intensities

Rather than computing features derived from the intensity histogram, it is also possible to use the whole set of histogram bins as feature vector. This normally results in a precise representation of the intensity distribution enabling a better discrimination of the foci versus the rest of the nucleus and measurement of the brightness ofboth.

#### Histogram of intensity surface curvature

The histogram of intensity surface curvature proposed in [3] is a histogram feature vector calculated on the intensity surface of the image. This histogram represents textural information, since local extrema of principal curvatures of the instensity surface describe foci or ridges, whereas homogeneous areas have very low curvature. The resulting feature vector is similar to the bag-of-gradients features [12], but is much more efficient to compute.

#### Haralick texture features

Different textures may have very similar intensity distributions, in which case they cannot be distinguished by the histogram features. To define a feature set that represents the actual texture, the local neighborhood must be considered. Haralick [13] described 14 features to classify textures, namely: angular second moment, contrast, correlation, sum of squares variance, inverse difference moment, sum average, sum variance, sum entropy, entropy, difference variance, difference entropy, two measures of correlation and max correlation coefficient. The features are calculated with the help of a gray level co-occurrence matrix (GLCM). The GLCM *P*_*d*,*θ*_ contains the normalized frequencies of gray levels of pixel pairs with the distance *d* in direction *θ*. It can be interpreted as probability of a neighborhood under this distance and direction. The intensity range is binned to reduce the influence of small differences. In order to achieve a rotation invariant feature set almost automatically without modifying the design of the underlying features, polar images (Section ‘Polar images’) are used in this work.

### Polar images

**Fig. 3:**
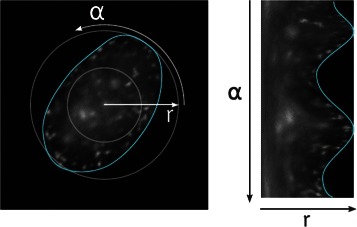
Polar image: Original image in Cartesian coordinates (left), polar image of the same region (right)

The approach followed in this work to convert directional features to rotation invariant features is to average them over a small number of directions. Polar images realize the concept of resampling the image to gain an advantage over the original representation. For this purpose, the normal Cartesian coordinates are mapped to polar coordinates. The resulting image displays the distance to the center on the *x* axis, and the angle on the *y* axis (Fig. 3). The angle is sampled with a high frequency. Depending on the size of the segment, the sampling is so frequent that all pixels in the segment are considered in the polar image, at least once.

It is straight forward to convert Haralick texture features, or any other directional feature, to rotation invariant features by simply calculating them on the polar image, with the *x* axis as direction. In this way the feature takes into account a lot more angles as compared to 4 directions as before, and is less demanding with regards to computational power.

### Classification

Classification is the process of learning a generalized model from a set of training data. Each training data consists of a feature vector, describing certain properties of the object, and the associated target class. From this input, the classifier learns a model which can be seen as function pointing from feature space to the target class value. The constructed model must be specific enough to classify the classes correctly.

In supervised learning, a human expert annotates the training samples with the target class values to get the ground truth needed for the learning process. The human expert has to be strict and label only the phases that can be clearly recognized in the given training dataset. If the labeling is carried out with care, the learned model will correctly distinguish between classes and patterns.

In the following, state-of-the-art classifiers are described, which were used in the evaluation of cell cycle phase differentiation.

#### Decision trees

The inner nodes of a Decision Tree are conditions based on a single attribute, while the branches correspond to the conditional cases of the node attribute, and leaf notes represent the class variable. Classification is achieved by traversing the tree starting at the root, following the matching conditional cases, down to a leaf node. The classification result is the label of the leaf node.

Training of Decision Trees is accomplished using the ID3 algorithm [14], which splits the training data set at that attribute where the split results in the highest information gain. The information gain is defined as entropy before minus the sum of entropy of all sets after the split. High information gain means that there is less entropy after the split, which means that the sets contain more similar objects. This split step is applied recursively to every new subtree and stops when the sets are unique. Decision trees are sensitive to overfitting though, which can be addressed by pruning the tree. This is accomplished by limiting the tree depth to a certain level or by stopping the learning process if the entropy drops below a certain value.

#### Support vector machines

Support Vector Machines (SVM) are binary classifiers that split up the feature space along a hyperplane $ \left (\vec w, b \right) $ with normal vector $\vec w$ and bias *b*. The result for the classification of sample $\vec x$ is indicated by the decision function $ c = \text {sign}\left (\left < \vec w, \vec x \right > + b \right) $, which refers to the position of the sample with respect to the hyperplane (i.e. above or below).

If the training data is linearly separable, many possible hyperplanes exist that classify the training data correctly. The best way to generalize to unseen data is to choose the hyperplane with the maximal margin to the training data. This results in the (eponymous) support vectors, parallel to the hyperplane, touching the nearest training data. The learning process is defined as optimization problem to construct the hyperplane with the maximal distance to the feature vectors. In order to classify not linearly separable training data, the slack variables *ξ*_*n*_≥0 are introduced. The hyperplane is calculated by minimizing

$${\frac{1}{2}} {||\vec{w}||}_{2}^{2} + \mathcal{C} \sum_{n=1}^{m} \xi_{n} $$

with respect to $\vec {w}$, under the side constraint $ c \left (\left < \vec {w}, \vec {x} \right > + b \right) \geq 1 - \xi _{n} $ for all $ \vec {x} $, with the tuning parameter $\mathcal {C} $. The optimization problem is solved using its dual representation.

A popular extension to handle not linearly separable training data is the kernel function. The idea is to map the feature space into a higher dimensional vector space, where the training data may be linearly separable. The kernel is a function so that *K*(*x*,*y*)=<*ϕ*(*x*),*ϕ*(*y*)> with *ϕ* the mapping from feature space to the higher dimensional vector space. This design allows to compute the inner product, which is needed in the dual representation, without mapping the *x* and *y* to the new space.

Classification problems with more than two classes are commonly addressed with the Error Correcting Coding Matrix (ECCM) approach. For each class, a distinct SVM classifies the membership versus all other classes. The ECCM defines bit strings encoding the original class values. A bit string of all binary classification results, obtained from classification based on the single class SVMs, is used to determine the final classification result. This is achieved by choosing the bit string with the minimal Hamming-distance.

#### Boosting

The idea of boosting is to build a strong classifier out of three weak classifiers and thus increase the classification rate. The strong classifier is defined as linear combination of weak classifiers, which are small decision units such as one-level decision trees.

Unlike the original approach with only three weak classifiers, the AdaBoost [15] method is capable of training an arbitrary number of weak classifiers. It performs an implicit feature selection by selecting in each iteration the feature with the highest improvement of the classification result. In this way, the feature space is reduced to the set of important features, saving computational time in the actual classification process.

It is possible to apply the ECCM approach mentioned in Section ‘Support vector machines’ to obtain a multi-class boosting classifier. However, there is a specially adapted version called AdaBoost.SIP [16]. Instead of constructing an ECCM with a strong classifier for each class, the ECCM contains a column for each learned weak classifier, defining an optimal class partition.

## Results and discussion

The presented approach was implemented using the software platform KNIME (The Konstanz Information Miner, www.knime.org), which already comprises feature sets and classification algorithms which could be employed in this work. The workflow was trained and evaluated on 79 images (0.5 GB) comprising 654 nuclei from the confocal and 843 nuclei from the widefield microscope (see Table 1). The workflow was executed on a computer with an Intel Xeon W3540 @2.93 GHZ and 6 GB memory, where the average computing time per image amounted to about 10 seconds.

**Table 1:** Sample sizes of cell classes of confocal and widefield microscopy images

class	Confocal	Widefield
G	354	616
Early S	138	166
Mid S	48	61
Late S	114	227
Total	654	843

The workflow was evaluated with respect to (1) segmentation accuracy, (2) feature performance, (3) classification accuracy for both confocal and widefield microscopy images. In the following Section ‘Segmentation’, the cluster splitting process is evaluated in comparison to the watershed algorithm. The evaluation of the different feature sets is presented in Section ‘Features’ and the performance of the different classifiers is considered in Section ‘Classification’. Finally, results for the different phases of the cell cycle, i.e. class-specific results, are provided in Section ‘Class-specific results’. The significance of comparing widefiled vs confocal microscopy is discussed in Section ‘Widefield vs. confocal microscopy segmentation’.

### Segmentation

**Fig. 4:**
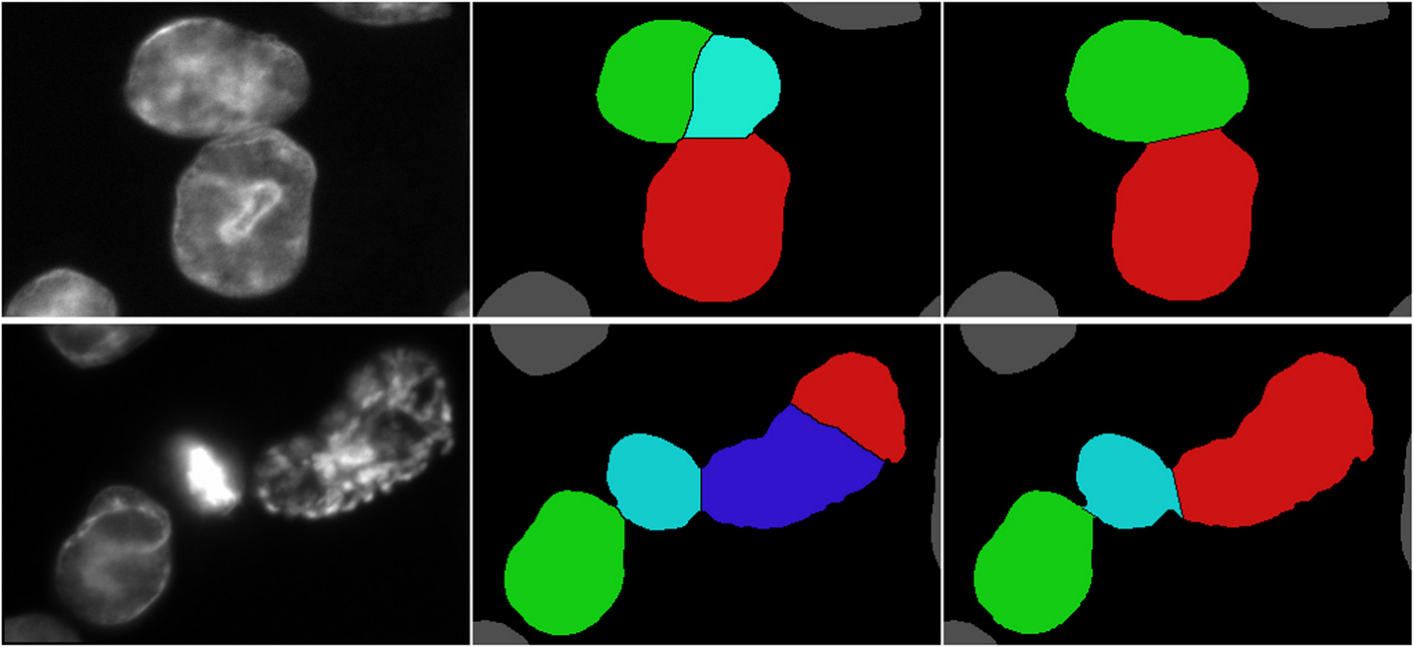
Comparison of segmentation approaches: Confocal image (left) and segmentation refined with the watershed method (middle). Same segments refined with the cluster splitting algorithm (right)

**Fig. 5:**
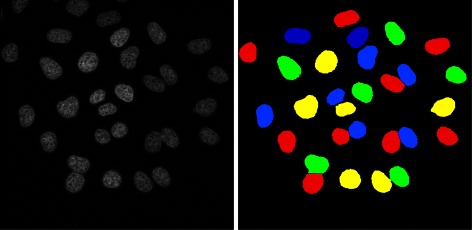
Microscopy image and final segmentation result: Original widefield input image (left) and processed image after segmentation and cluster splitting (right)

The segmentation result of the proposed cluster splitting algorithm was evaluated against the manually annotated nuclei and compared to the classical watershed algorithm. Figure 4 shows the segmentation result of both methods for interesting cell clusters in a confocal microscopy image. An entire widefield microscopy image and the corresponding final segmentation result is shown in Fig. 5.

In order to evaluate appropriate splitting of the clusters, the point annotation providing the target classes can be used to distinguish correctly from erroneously split clusters. The measurements used to evaluate the segmentation were the probability of correctly segmented, over-segmented and under-segmented nuclei.

The probability of over-segmented nuclei is the number of fragments without a matching annotation divided by the total number of nuclei. The probability of under-segmented nuclei is the number of nuclei with more than one matching annotation divided by the total number of nuclei. The probability of correctly segmented nuclei is defined as 1−*over-segmented*−*under-segmented*. Table 2 shows the performance of the segmentation process.

**Table 2:** Segmentation performance of the cluster splitting algorithm versus the watershed method

	Confocal		Widefield	
	Cluster	Watersh.	Cluster	Watersh.
Correct	0.864	0.671	0.847	0.811
Over-seg.	0.12	0.281	0.15	0.183
Under-seg.	0.016	0.048	0.003	0.006

Both methods show a high percentage of correctly segmented cell nuclei. The watershed method with its excessive splitting strategy shows a much high over segmentation. Up to 28 % of the nuclei are unnecessarily split. In comparison, the worst case for the developed cluster splitting is 15 % over segmentation.

Furthermore, the watershed methods leaves up to 4.8 % of the segments not completely decomposed, while the cluster splitting reduces the amount of remaining clusters to 1.6 %.

In summary, these data show that the geometrical cluster splitting is able to avoid unnecessary splits (which the watershed method would perform) and at the same time splits clusters the watershed method would ignore.

However, it should be noted that the methods and parameters in the proposed semi-automated pipeline were chosen to provide correct segmentation results on the cell images processed in this work. The actual preprocessing steps and parameter settings also highly depend on the biological sample, hence other biological images may require different preprocessing steps. Further options for preprocessing of cell images are available through e.g. ImageJ [17] or CellProfiler [18].

### Features

To evaluate the effectiveness of the feature sets, a 10-fold cross validation was performed. After a z-score normalization, the data was divided in 10 pairs of training and test sets using random sampling. For classification, the KNIME decision tree predictor was used which performed about 10 % better than the support vector machine.

The following features were considered:

1. Histogram features: This feature set comprises the basic histogram features (Min, Max, Mean, Variance, Skewness, Kurtosis).
2. Haralick texture features: This is a 104 dimensional feature vector, that contains all Haralick texture feature calculated on two vertical stripes of the polar, in horizontal and vertical direction.
3. Histogram of intensities: This feature set is a 64 bin histogram of intensity.
4. Histogram of curvatures: This feature set is a 64 bin histogram of surface curvature.

The combination of histogram features and Haralick features (1 and 2) forms the feature set proposed in this work according to measured performance. The feature vector proposed by [3] is equivalent to the combination of feature 3 and 4.

**Fig. 6:**
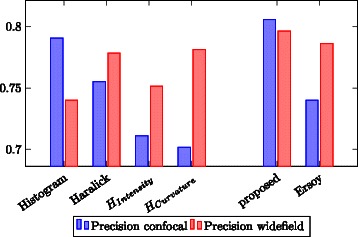
Feature performance: Classification accuracy for the four semantic feature groups (basic histogram features, Haralick texture features, histogram of intensity, histogram of surface curvature) and both feature sets

Performance of the feature groups was measured using the classification accuracy. Figure 6 shows the accuracy for all four feature groups and both combined feature sets.

The basic histogram features (feature group 1) show a high accuracy especially on the confocal images. Despite the small number of features in this group, the accuracy for the confocal images is the highest of all four feature groups. Haralick texture features achieve a good accuracy and proved to be robust with respect to the quality differences of the image source. The proposed feature set, a combination of histogram features and Haralick texture features, shows a high accuracy and low variation between confocal and widefield microscopy images.

On widefield images, feature groups 3 and 4 are comparable to the first two feature groups. In the case of confocal images, both show a very low accuracy, which is also true for their combination. I.e. the feature set proposed by [3] proved to be less robust than the feature set suggested in this work.

### Classification

**Fig. 7:**
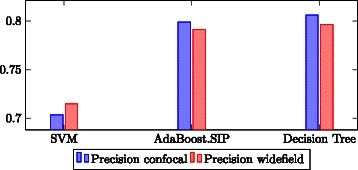
Classifier performance: Classification accuracy for all the classifiers on the proposed feature set

In order to determine the best classification algorithm, the workflow was trained on the proposed feature set using the same 10-fold cross validation setup as in the feature evaluation (Section ‘Features’). Each classifier presented in Section ‘Classification’ was trained and evaluated. Figure 7 shows the classification accuracy. Due to the high hardware requirements of the frequent item set mining of the AdaBoost.SIP classifier, it was performed on four equally sized subsets of the feature vector. In this way, it was possible to run the evaluation of the AdaBoost.SIP classifier on the test PC. However, by running the AdaBoost.SIP classifier on a subspace of the available features, not all feature combinations are possible. For this reason, the AdaBoost.SIP classifier may perform better after eliminating these hardware limitations.

We had initially expected that SVM would perform well, since good classification results based on SVM have been reported in the literature [3–5]. However, it should be noted that their sample sizes are much higher. Since SVM are known to need relatively large training data to perform well, the smaller amount of training data may give rise to the performance result of SVM in comparison to the other classifiers. However, since manual annotation of training data is an extremely time consuming task for biologists, it should be emphasized that a classifier is required that performs well on a limited amount of data.

Even though not significantly better than the decision tree in terms of classification results, the AdaBoost.SIP takes up to more than an hour for the learning process, whilst the decision tree runs in only about one minute. For this reason, the decision tree classifier was used to perform all further evaluations.

It should be noted that only the most common and popular classification algorithms were investigated in this work. There are many more classification algorithms available in the literature, such as Kernel estimation methods, different variants of Boosting, Decision Trees (as well as Random Forests), or Neural Networks. Also, a multitude of open-source classification frameworks is available, which could have alternatively been used for implementation. They are available either as generic frameworks (such as WEKA [19] or KNIME [20]) or integrated into bioanalysis tools (such as PSLID/SLIC [21], CellProfiler [18], wndchrm [22], CellExplorer [23], ilastik [24] or BIOCAT [25]), please see [26] for an overview.

### Class-specific results

**Fig. 8:**
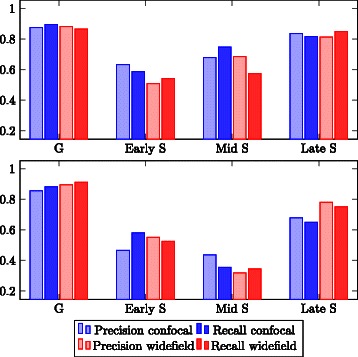
Class-specific precision and recall. Top: Suggested feature set (combined features 1 and 2). Bottom: Feature set (combined features 3 and 4) proposed by Ersoy [3]

This section evaluates the goodness of the classification achieved using the proposed feature set and the decision tree classifier. The measurements used to perform this class level evaluation are precision and recall, which are defined as follows:

$${precision}_{C} = \frac{tp}{tp+fp}, {recall}_{C} = \frac{tp}{tp+fn}, $$

where *tp* (true positive) is the number of cells correctly classified as class *C*, *fp* (false positive) is the number of cells erroneously classified as class *C* and *fn* (false negative) is the number of cells belonging to class *C* but classified otherwise. Overall accuracy is the number of correctly classified cells divided by the total number of cells. Figure 8 shows precision and recall for the suggested feature set, and the feature set proposed by [3].

In general, early S and mid S phase are distinguished at a much lower precision than G and late S phase. The low precision of recognizing early S phase cells is due to the very similar pattern of PCNA localization in this phase and the G phases. This effect is influenced by the convolution of the images occurring in the microscope and is therefore more pronounced in the widefield images, while the confocal images show a more distinct early S phase pattern. As for the mid S phase, there were only very few cells in the population showing this pattern.

The results for the feature set proposed by [3] show the same tendencies. However, in comparison to the proposed feature set, precision and recall for the recognition of mid S phase are strongly decreased and for late S phase slightly decreased.

For this reason, it can be concluded that the suggested feature set, which combines histogram features and Haralick texture features, allows a better discrimination of mid S and late S phase.

### Widefield vs. confocal microscopy segmentation

Though there are no easily accessible statistics concerning the numbers of widefield vs confocal systems installed in life science laboratories, it is obvious that widefield systems are much more widespread due to their comparably low cost and ease of operation.

Widefield microscopy is always the technique of choice for very light sensitive samples as image acquisition is much faster. For 3D spatial resolution it can be combined with deconvolution yielding results almost identical to confocal microscopy. Exact knowledge of the point spread function of the imaging system, which is not always easily available, is necessary for obtaining accurate results. Though fast deconvolution algorithms have been developed enabling processing “on the fly”, they still represent an additional step. This is one of the reasons why many biology laboratories heavily rely on confocal microscopy, in particular for colocalization analysis.

In order to provide an image analysis tool of general utility it is thus important to demonstrate its feasibility for images acquired with either method, widefield and confocal. As shown and discussed in Sections ‘Features’ and ‘Class-specific results’, the proposed pipeline performs equally well for both microscopy techniques.

## Conclusion

In this work, an optimal processing pipeline was devised and evaluated with respect to segmentation accuracy, performance of features and classification. By taking into account images from both confocal and widefield microscopes, it was also possible to compare the influence of the image source on the overall classification quality of different cell cycle phases.

Overall, it could be shown that the developed workflow is capable of classifying the cell cycle stages in PCNA-immunolabeled cells from single time point images at a high quality, which is of great value to the biologists compared to manual evaluation.
